# Characterization of the complete chloroplast genome of *Firmiana hainanensis* (Malvaceae), an endemic and vulnerable tree species of China

**DOI:** 10.1080/23802359.2022.2160669

**Published:** 2023-01-02

**Authors:** Fengxiao Tan, Achyut Kumar Banerjee, Jie Deng, Hui Feng, Yuanjiao Feng, Yinghua Shu, Jianwu Wang

**Affiliations:** a Guangdong Provincial Key Laboratory of Eco-Circular Agriculture, College of Natural Resources and Environment, South China Agricultural University, Guangzhou, PR China; b School of Life Sciences, Sun Yat-sen University, Guangzhou, PR China; c School of Aeronautics and Astronautics, Sun Yat-sen University, Shenzhen, PR China

**Keywords:** Firmiana, chloroplast genome, endemic tree, phylogeny, vulnerable species

## Abstract

*Firmiana hainanensis* Kosterm. is a commercially valuable endemic tree species in China and has long been considered a globally vulnerable species. We assembled and characterized the complete chloroplast genome of this species by using Illumina pair-end sequencing data. The total chloroplast genome size was 161,559 bp, including two inverted repeats (IRs) of 25,612 bp, separated by a large single copy (LSC) and a small single copy (SSC) regions of 90,057 and 20,277 bp, respectively. A total of 130 genes were identified, including 85 protein-coding genes, 37 *tRNA*, and eight *rRNA* genes. Phylogenetic analysis showed that *F. hainanensis* was the most basal species in the genus *Firmiana*. The chloroplast genome of this species will provide a theoretical basis to understand the taxa’s evolution further and is expected to contribute to its conservation efforts.

## Introduction

**Figure 1.**
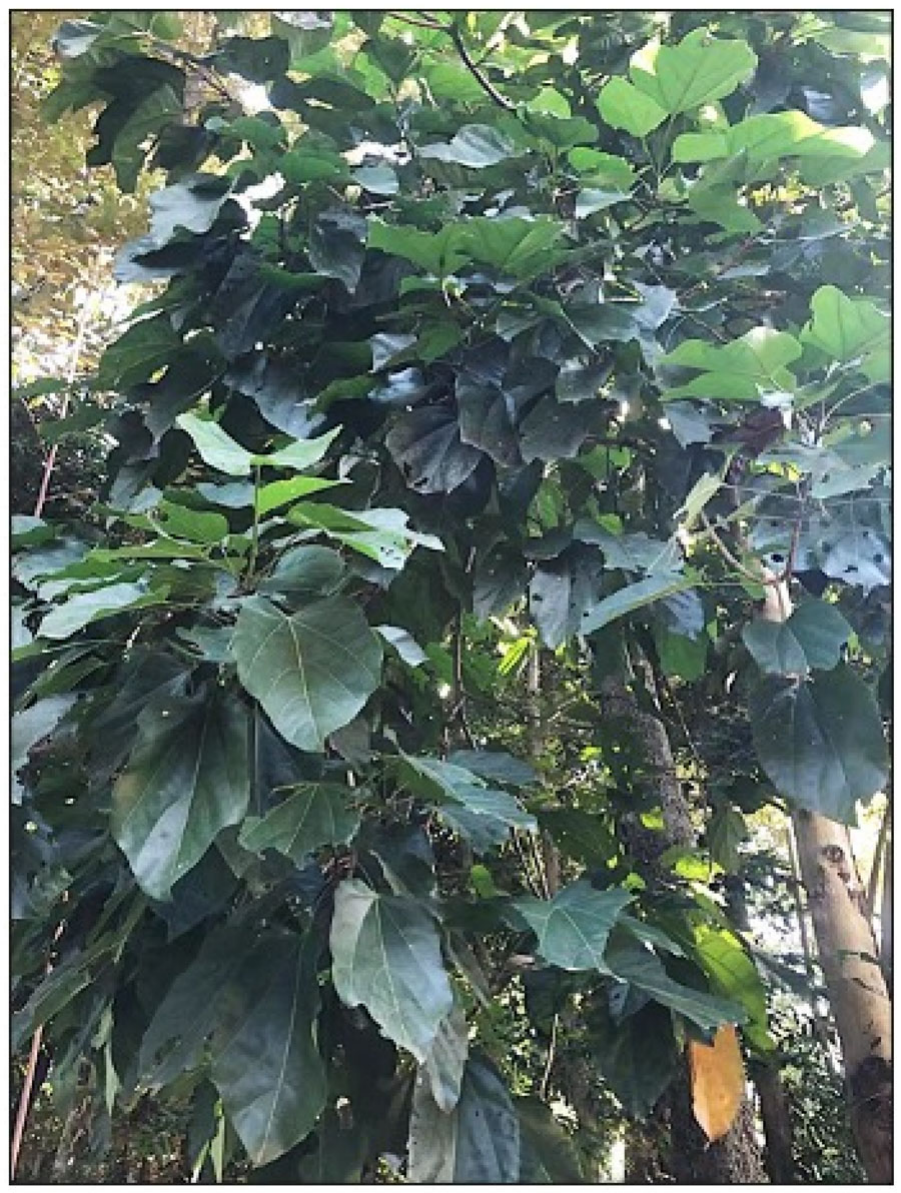
*Firmiana hainanensis* Kosterm., a commercially valuable endemic tree species in China. The photo was taken at the South China Botanical Garden, Guangzhou, China.

The genus *Firmiana* consists of 12-16 species worldwide (Tang et al. 2007). Seven species of this genus are found in China, of which five are endemics with narrow distribution ranges (Chen et al. 2014). Among these species, *Firmiana hainanensis* Kosterm. 1956 (Figure 1) is the most southernly distributed species and is found only in the mountain areas of central and southern Hainan (Tang et al. 2007). The species has multiple economic values in the forms of raw materials for paper and rope production, furniture making, and ornamental uses. The species has long been listed as a vulnerable species globally (https://www.iucnredlist.org/species/32405/9703111; accessed on 30 July 2022). A better insight into genomics helps us to understand the evolutionary history of the species leading to better conservation efforts. The chloroplast genomes (plastomes, hereafter) have uniparental inheritance, relatively conserved structure, and are often characterized by low recombination and substitution rate (Twyford and Ness 2017). Therefore, complete sequences of plastomes have often been used to study plant evolution [e.g. (Wei and Li 2022)]. Plastome sequences are increasingly available for many plant taxa (Daniell et al. 2016), yet such accounts are rare for the species of the genus *Firmiana*. To fill the knowledge gaps and assist in ongoing and future conservation efforts, we assembled and characterized the complete chloroplast genome of *F. hainanensis* in this study.

## Materials

The fresh leaves of an individual of *F. hainanensis* were collected from South China Botanical Garden, Chinese Academy of Sciences, Guangzhou, Guangdong Province, China (Longitude: 113.3682 E, Latitude: 23.1821 N) for genomic DNA extraction. The voucher specimens were deposited in the herbarium of Sun Yat-sen University with the voucher number: Jian 20965 (contact: Hui Feng, fengh35@mail2.sysu.edu.cn).

## Methods

Total genomic DNA was extracted by a modified CTAB method (Allen et al. 2006). The 350 bp paired-end library was constructed according to the manufacturer’s introductions (Illumina) and sequenced on the Illumina Novaseq 6000 platform by Berry Genomics Company (http://www.berrygenomics.com/; Beijing, China). A total of 15 GB 150 bp paired-end raw reads were retrieved. Then, quality-trimmed clean reads were used for the chloroplast genome *de novo* assembly using GetOrganelle with default parameters (Jin et al. 2020). The assembly was further evaluated using BWA-MEM version 0.7.17-r1188 (Li 2013) and QualiMap version 2.2.2-dev (Okonechnikov et al. 2016), respectively. The mean coverage depth is 302 X (Figure S1). The chloroplast genome of *F. hainanensis* was annotated with the GeSeq automatically (Tillich et al. 2017). A final circular chloroplast genome map was drawn using OGDRAW (Greiner et al. 2019).

To understand the phylogenetic position of *F. hainanensis* in the family Malvaceae (Angiosperm Phylogeny Group 2016), we downloaded 26 published chloroplast genomes from the NCBI GenBank database. From the family Malvaceae, we downloaded chloroplast genomes of 24 species, out of which 11 species belong to the subfamily Sterculioideae. Among these 11 species, seven belong to the genus *Firmiana*. Two species, *Phaleria macrocarpa* and *Gonystylus affinis* in the family Thymelaeaceae, were considered as the outgroup. Multiple sequences alignment was achieved by HomBlocks pipelines (Bi et al. 2018). The maximum-likelihood tree (bootstrap replications 1000), inferred by the best-fit model of GTR + F + I + G4, was constructed using IQ-TREE version 2.0.3 (Minh et al. 2020).

## Results

**Figure 2.**
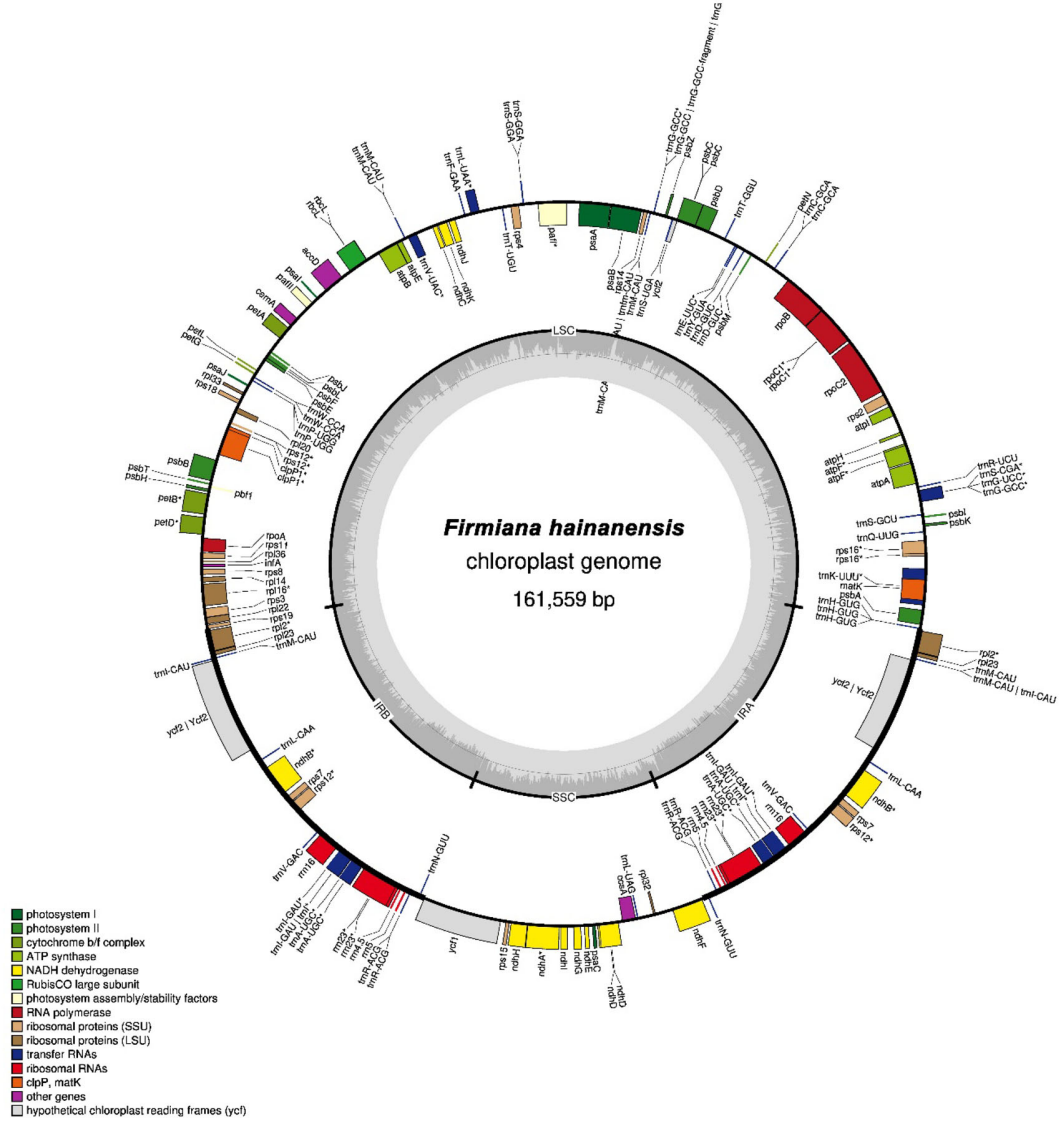
The chloroplast genome map of *Firmiana hainanensis* showing the regions of 130 genes. The inner circle of darker grey color represents the Guanine and Cytosine (GC) content, and of lighter grey color represents the Adenine and Thymine (AT) content of the chloroplast genome. The genes drawn outside and inside of the outer circle are transcribed counter clockwise and clockwise, respectively. LSC: Large single copy, SSC: Small Single Copy; IR (IRA and IRB): Inverted Repeats. The functional groups of the genes are shown in different colors.

The circular chloroplast genome of *F. hainanensis* was found to be 161,559 base pairs (bp) long having 36.75% GC content (Figure 2). In the genome, the large single copy (LSC) region was of 90,057 bp long, whereas the length of the small single-copy (SSC) region was 20,277 bp. The LSC and SSC regions were separated by two inverted repeats (IRs), each having 25,612 bp. The genome contained 130 genes, consisting of 85 protein-coding genes, 37 *tRNA* genes and eight *rRNA* (*5S rRNA*, *4.5S rRNA*, *23S rRNA*, and *16S rRNA*) genes. Six of the protein-coding genes, eight of the tRNA genes, and four of the *rRNA* genes were found to be duplicated in the IRs. The complete chloroplast genome sequence has been submitted to GenBank (accession number ON813240).

**Figure 3.**
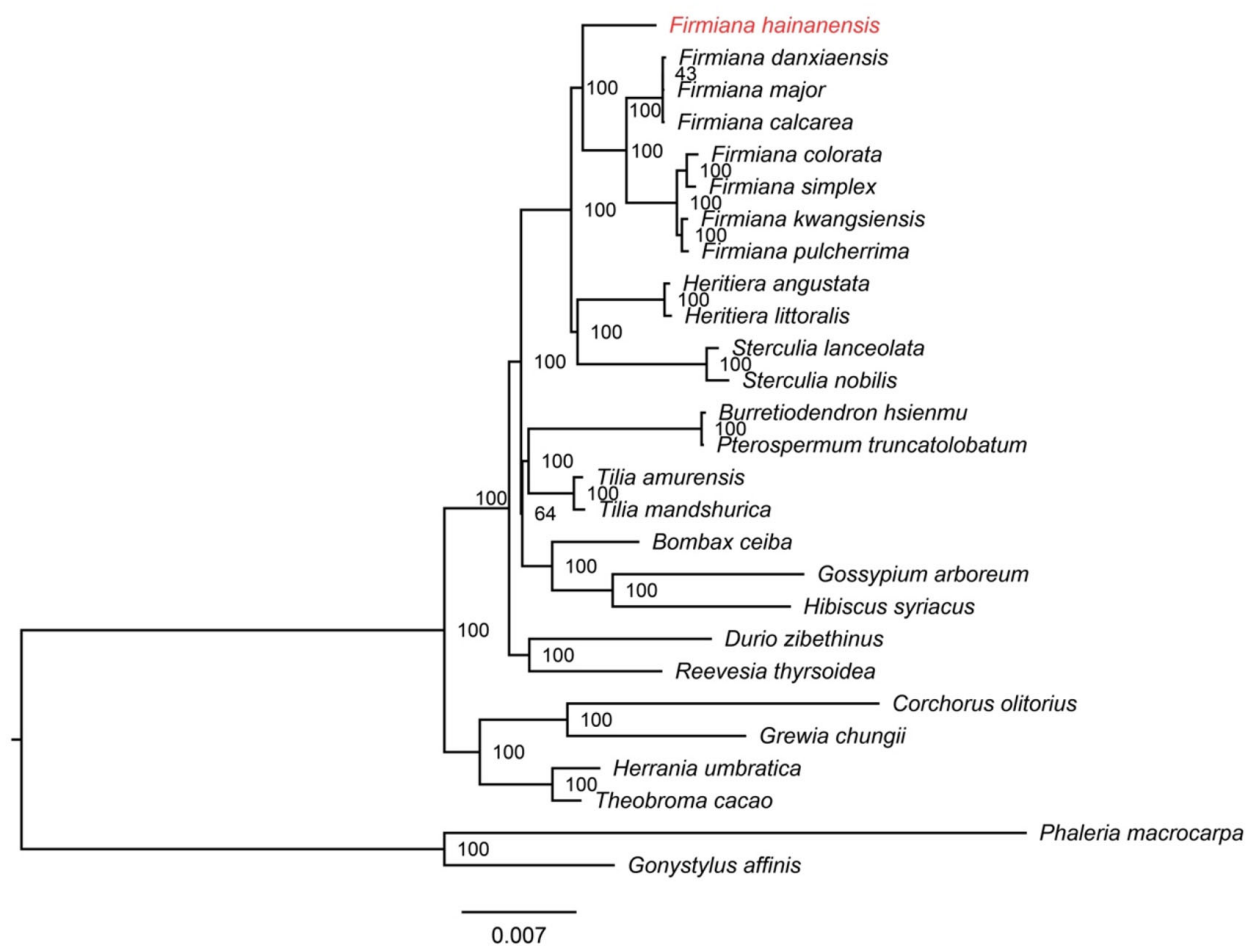
Maximum-likelihood tree based on the sequences of 27 complete chloroplast genomes. Numbers in the nodes are bootstrap support values from 1000 replicates. The position of *Firmiana hainanensis* is shown in red. The GenBank accession numbers for each species and the citation sources for those published sequences are provided in Table S1.

The phylogenetic relationships among the 27 species (*F. hainanensis*, 24 Malvaceae species and two Thymelaeaceae species as outgroups) were supported by high bootstrap values (Figure 3). The phylogeny showed that *F. hainanensis* was the earliest diverging species in the genus *Firmiana*.

## Discussion and conclusion

The chloroplast genome of *F. hainanensis* was characterized for the first time in this study. Our findings will add new insights into the growing evidence of complete plastome sequences of angiosperm taxa. The plastome length of *F. hainanensis* is comparable to other Malvaceae species, e.g. *Reevesia pycnantha* (Zhang et al. 2022), but is shorter than Durian (*Durio zibethinus* L.; Se-Hwan et al. 2017). The findings will provide a theoretical basis to understand the taxa’s evolution further and is expected to contribute to its conservation efforts.

## Supplementary Material

Supplemental Material

Supplemental Material

## Data Availability

The *Firmiana hainanensis* genome sequence data are available in GenBank under accession number ON813240. The associated BioProject, SRA, and Bio-Sample numbers are PRJNA850651, SRR19769910, and SAMN29200181, respectively.
