# Supplementary material for: Characterization of the complete chloroplast genome of Firmiana hainanensis (Malvaceae), an endemic and vulnerable tree species of China
Source: Mitochondrial DNA B Resour. 2023 Jan 2;8(1):57–60. doi: 10.1080/23802359.2022.2160669 (PMC9817124; doi:10.1080/23802359.2022.2160669)
Supplement: Supplemental Material [file TMDN_A_2160669_SM3716.docx]

| 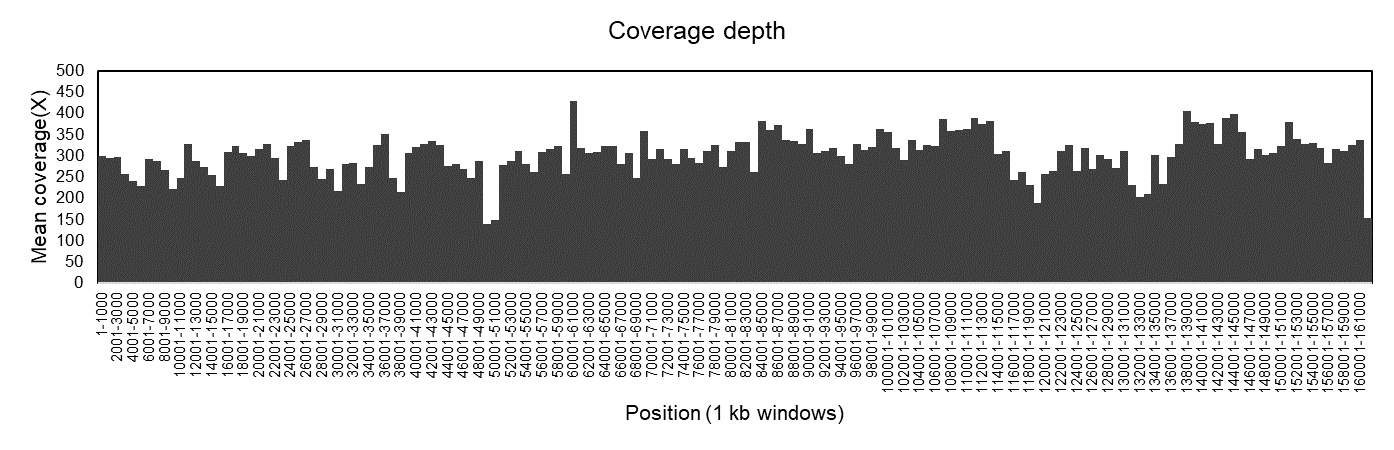 |
| --- |
|  |

Figure S1: The coverage depth for chloroplast genome of *Firmiana hainanensis* using 1kb windows based on the analysis results using BWA-MEM 0.7.17-r1188 and Samtools v1.7. The mean coverage depth is in the range 138 to 429, and the mean coverage depth of the complete genome sequence were 302 X.
