# Supplementary material for: Characterization of the complete chloroplast genome of Firmiana hainanensis (Malvaceae), an endemic and vulnerable tree species of China
Source: Mitochondrial DNA B Resour. 2023 Jan 2;8(1):57–60. doi: 10.1080/23802359.2022.2160669 (PMC9817124; doi:10.1080/23802359.2022.2160669)
Supplement: Supplemental Material [file TMDN_A_2160669_SM3715.docx]

| Table S1. The accession numbers for all 27 species used in the phylogeny reconstruction and the citation sources for those sequences published | | |
| --- | --- | --- |
| Species name | Accession number | Reference |
| *Bombax ceiba* | NC_037494 | [1] |
| *Burretiodendron hsienmu* | MN533963 | [2] |
| *Corchorus olitorius* | MK251465 | - |
| *Durio zibethinus* | NC_036829 | [3] |
| *Firmiana calcarea* | MN826071.1 | - |
| *Firmiana colorata* | MN533965 | [2] |
| *Firmiana danxiaensis* | MN480318.1 | [4] |
| *Firmiana hainanensis* | ON813240 | This study |
| *Firmiana kwangsiensis* | MN786867.1 | [5] |
| *Firmiana major* | NC_037242 | - |
| *Firmiana pulcherrima* | NC_036395 | [6] |
| *Firmiana simplex* | MN533966 | [2] |
| *Gonystylus affinis* | NC_052860.1 | [7] |
| *Gossypium arboreum* | HQ325740 | [8] |
| *Grewia chungii* | MN533967 | [2] |
| *Heritiera angustata* | NC_037784 | - |
| *Heritiera littoralis* | MK033518 | [9] |
| *Herrania umbratica* | MN163033 | - |
| *Hibiscus syriacus* | KP688069 | - |
| *Phaleria macrocarpa* | NC_052861.1 | [7] |
| *Pterospermum truncatolobatum* | MN533971 | [2] |
| *Reevesia thyrsoidea* | NC_041441 | [10] |
| *Sterculia lanceolata* | MN533973.1 | [2] |
| *Sterculia nobilis* | MZ958831.1 | - |
| *Theobroma cacao* | NC_014676 | [11] |
| *Tilia amurensis* | NC_028588 | [12] |
| *Tilia mandshurica* | NC_028589 | [12] |

Reference

1. Gao Y, Wang H, Liu C, Chu H, Yan Y, Tang L. 2018. Complete chloroplast genome sequence of the red silk cotton tree (*Bombax ceiba*). Mitochondrial DNA B Resour; 3(1):315-316.
2. Wang JH, Moore MJ, Wang H, Zhu ZX, Wang HF. 2021. Plastome evolution and phylogenetic relationships among Malvaceae subfamilies. Gene; 765:145103.
3. Cheon SH, Jo S, Kim HW, Kim YK, Sohn JY, Kim KJ. 2017. The complete plastome sequence of Durian, *Durio zibethinus* L. (Malvaceae). Mitochondrial DNA B Resour; 2(2):763-764.
4. Lu Q, Luo W, Huang Z. The complete chloroplast genome of *Firmiana danxiaensis*, an endangered species endemic to Danxia landform in Southern China. 2019. Mitochondrial DNA B Resour; 4(2):4071-4072.
5. Lu Q, Luo W. 2022. The complete chloroplast genome of two *Firmiana* species and comparative analysis with other related species. Genetica; 150(6):395-405.
6. Zhao KK, Wang JH, Cai YC, Zhu ZX, López-Pujol J, Wang HF. 2018. Complete chloroplast genome sequence of *Heritiera angustata* (Malvaceae): an endangered plant species. Mitochondrial DNA B Resour; 3(1):141-142.
7. Hishamuddin MS, Lee SY, Ng WL, Ramlee SI, Lamasudin DU, Mohamed R. 2022. Comparison of eight complete chloroplast genomes of the endangered Aquilaria tree species (Thymelaeaceae) and their phylogenetic relationships. Sci Rep; 10(1):13034.
8. Xu Q, Xiong G, Li P, He F, Huang Y, Wang K, Li Z, Hua J. 2012. Analysis of complete nucleotide sequences of 12 *Gossypium* chloroplast genomes: origin and evolution of allotetraploids. PLoS One; 7(8): e37128.
9. Shi C, Han K, Li L, Seim I, Lee SM, Xu X, Yang H, Fan G, Liu X. 2020. Complete Chloroplast Genomes of 14 Mangroves: Phylogenetic and Comparative Genomic Analyses. Biomed Res Int; 2020:8731857.
10. Quan G, Zou P, Liu G, Sun M, Wang W, Dai S. 2019. The complete chloroplast genome of *Reevesia thyrsoidea* (Malvaceae). Mitochondrial DNA B Resour; 5(1):292-293.
11. Kane N, Sveinsson S, Dempewolf H, Yang JY, Zhang D, Engels JM, Cronk Q. 2012. Ultra-barcoding in cacao (*Theobroma* spp.; Malvaceae) using whole chloroplast genomes and nuclear ribosomal DNA. Am J Bot; 99(2):320-9.
12. Cai J, Ma PF, Li HT, Li DZ. 2015. Complete Plastid Genome Sequencing of Four *Tilia* Species (Malvaceae): A Comparative Analysis and Phylogenetic Implications. PLoS One; 10(11): e0142705.
